# Impact of Cardiac Dose on Overall Survival in Lung Stereotactic Body Radiotherapy (SBRT) Compared to Conventionally Fractionated Radiotherapy for Locally Advanced Non-Small Cell Lung Cancer (LA-NSCLC)

**DOI:** 10.4236/jct.2021.127036

**Published:** 2021-07

**Authors:** Justin D. Anderson, Jiuyun Hu, Jing Li, Steven E. Schild, Mirek Fatyga

**Affiliations:** 1 Department of Radiation Oncology, Mayo Clinic, Phoenix, Arizona, USA; 2 School of Computing, Informatics, and Decision Systems Engineering, Tempe, Arizona, USA; 3 School of Industrial and Systems Engineering, Georgia Institute of Technology, Atlanta, Georgia, USA

**Keywords:** Lung Cancer, Lung SBRT, Cardiac Toxicity, Lung Radiation Therapy, Non-Small Cell Lung Cancer

## Abstract

**Purpose:**

To examine possible association between heart irradiation and Overall Survival (OS) in lung SBRT patients and to compare observed associations with cardiac toxicity models previously derived in LA-NSCLC patient studies.

**Materials and Methods:**

197 Patients treated with lung SBRT at Mayo Clinic Arizona were selected for this IRB-approved study. Multivariate Cox model with Akaike Information Criterion (AIC) was used to select patient specific covariates associated with OS. Heart dosimetry was represented by *V_D_* indices, which is a percentage of volume exposed to dose D or greater. Multivariate Cox models with patient specific covariates and single *V_D_* index per model was used to find a range of doses which were predictive for OS. A digital subdivision of the heart was further used to determine the spatial distribution of doses which were predictive for OS. A coarse subdivision divided heart into 4 segments, while the fine subdivision divided heart into 64 segments. Knowledge constrained Fused Lasso operator was used to derive a more complete model which correlated heart dosimetry with OS. Results of statistical analysis were compared to predictions of a model of cardiac toxicity in LA-NSCLC patients.

**Results:**

Higher age (p < 0.001), higher stage (p < 0.001) and squamous cell histology (p = 0.001) were associated with reduced OS. Whole heart DVH analysis did not reveal associations between heart irradiation and reduced OS. Coarse subdivision of the heart into four segments revealed that the irradiation of two inferior segments of the heart with low doses was associated with reduced OS, *V*_2*Gy*_ in the right-inferior segment (HR = 1.012/1%, p = 0.02), and *V*_1*Gy*_ in the left-inferior segment (HR = 1.01/1%, p = 0.04). Maximum dose in the right-inferior segment of the heart was also associated with reduced OS (HR = 1.02/Gy, p = 0.02). Fine subdivision of the heart into 64 segments revealed that approximately 25% of heart volume in the inferior part of the heart (15/64 segments), when irradiated to doses in the 1 Gy - 5 Gy range, were predictive for reduced OS (HR = 1.01/1%, p = 0.01). A previously derived model of cardiac toxicity in LA-NSCLC patients did not predict a reduction of OS due to heart irradiation in lung SBRT patients, because of relatively low doses to the heart in most lung SBRT patients.

**Conclusions:**

Doses lower than 5 Gy in the inferior segments of the heart may be associated with reduced overall survival in patients treated for lung lesions with SBRT. Stage and histology of the disease, as well as patients’ age, were also associated with overall survival. Comparisons of cardiac toxicity patterns in LA-NSCLC patients and lung SBRT patients suggest different etiology of cardiac toxicity in the two groups.

## 1. Introduction

Late cardiac toxicity effects which typically occur years to decades after Radiation Therapy (RT) have been well documented [1]. Clinical strategies for avoiding late cardiac toxicity, like Deep Inspiration Breath Hold during RT (DIBH), are commonly used in clinical practice [2].

More recent, prospective studies suggested that radiation induced cardiac toxicity can be more significant than previously thought and may occur within one to two years after RT. Radiation Therapy Oncology clinical trial RTOG 0617 compared standard dose (60 Gy) versus high dose (74 Gy) radiation with concurrent chemotherapy (cetuximab) in LA-NSCLC patients. The high dose arm of RTOG 0617 was closed early because of the observed reduction in OS, when compared to the conventional dose arm. Subsequent analysis of RTOG 0617 associated increased heart *V*_40_ [3] and heart *V*_50_ [4] with decreased OS, offsetting any potential advantages due to higher local control of the disease. Additional studies associated irradiation of superior structures of the heart with cardiac events and reduced OS in NSCLC patients [5] [6] [7] [8] [9]. Limited life expectancy of LA-NSCLC patients suggested that cardiac damage was significant and occurring shortly after treatments. A recent retrospective study of death certificates of NSCLC patients further suggested that cardiovascular death could be significantly under-reported in this patient population [10]. Treatment planning studies which seek to limit heart irradiation in LA-NSCLC patients have been recently reported [11] [12].

Earlier stages of lung cancer or small metastatic lung lesions are treated with Stereotactic Body Radiation Therapy (SBRT). SBRT is limited to small tumor sizes which implies that large physical doses to the heart should be relatively uncommon, but it is also hypo fractionated which could increase biological damage to the heart. Longer life expectancy of patients treated for earlier stage lung cancer could reveal new etiologies of cardiac toxicity which were not visible in LA-NSCLC patient populations. Cardiac toxicity in lung SBRT has not been extensively studied and published findings present an ambiguous picture, with two studies suggesting cardiac toxicity due to heart irradiation [13] [14] and two studies reporting no statistically significant cardiac toxicity [15] [16].

The purpose of this work was to examine possible associations between heart irradiation and OS in lung SBRT patients and to compare the findings to similar associations observed in LA-NSCLC patients. The present study used a novel methodology of digital subdivision of the heart, previously developed for studies of cardiac toxicity in LA-NSCLC patients [8], enabling a comparison of cardiac toxicity in the two patient populations.

## 2. Materials & Methods

### 2.1. Patients

IRB approval was obtained to perform a retrospective analysis of patients treated with lung SBRT at our institution. Patients were treated between 2010 and 2020 using a combination of static field IMRT and VMAT techniques. Only patients who had a single SBRT treatment were included in the study. Patient characteristics are presented in Table 1. Institutional Tumor Registry was used to obtain survival data.

### 2.2. Dosimetry

Treatment plans were created using Eclipse Treatment Planning System (Varian, Inc) and doses were calculated using the AAA algorithm. Dosimetric information was extracted from Eclipse using Application Programming Interface (API) and processed further using in-house software. Doses were projected from the dose grid on the 3D structure of organs of interest and recorded in a format which preserved 3D dose distribution within each organ. Physical dose was converted to 2 Gy equivalent dose for each organ, voxel by voxel, using linear-quadratic model and organ specific alpha over beta ratios (heart $\frac{\alpha }{\beta }=2$; lung $\frac{\alpha }{\beta }=3$). Converted doses were used to form Dose Volume Histograms (DVH) and for the analysis of correlations between 3D dose distributions and clinical outcomes.

### 2.3. Statistical Analysis

Statistical analysis was performed in stages.

#### 2.3.1. Selecting Patient Specific Covariates

Multivariate Cox model with Akaike Information Criterion (AIC) was used to select patient specific covariates which were associated with OS. Heart dosimetry was excluded from this stage of the analysis. Age, gender, stage of the disease, histology, tumor laterality, mean lung dose and median lung dose were included as candidate covariates.

#### 2.3.2. Adding Heart DVH Covariates to the Analysis

##### 1) Univariate Analysis

Heart dosimetry was represented by a *V_D_* index which is the percentage of the volume of the heart receiving dose D, or greater. A family of multivariate Cox models was fit, each model including all patient specific covariates that were found to be predictive and one *V_D_*. The value of dose D was changed to effectively scan the space of *V_D_* indices. In each fit the p-value associated with the *V_D_* variable was obtained to establish a range of doses which were predictive for OS, using the criterion of p < 0.05.

##### 2) KC-Lasso Model

Once the dose range has been established a linear predictor composed of an array of *V_D_* indices within the range, *h*(*t*) = *h*_0_ (*t*)exp(*β*_0_ + *β*_1_*V*_*D*1_ + ⋯ + *β_p_V_Dp_*), was constructed. To account for the high degree of correlations between *V_D_* coefficients we took advantage of modern developments in statistical analysis by adding constraints on the coefficient estimates which are known as “variable selection techniques” [17]. Specifically, Fused Lasso [18] [19] model was used which imposes L1 penalties on the coefficient estimates, $\sum_{i=1}^{p}|\beta_{i}|<s$, and on adjacent dose levels, $\sum_{i=1}^{p}|\beta_{i}-\beta_{i-1}|<\gamma$, having the effect of suppressing small-effect coefficients and accounting for high degree of correlations among dosimetric variables. The upper bounds in the constraints, *s* and *γ*, are selected using a grid search to optimize a commonly used model selection criterion. Following the work of Dai and Breheny [20] we incorporated leave-one-out cross validation of linear predictors during the grid search to find parameters *s* and *γ* which were associated with the lowest cross validation error. In this analysis we also imposed two additional constraints on coefficient estimates which reflected radiobiological considerations. The positivity constraint prohibits negative coefficients since tissue irradiation should never reduce the risk of toxicity. The monotonicity constraint requires that *β*_1_ ≤ *β*_2_ ≤ ⋯ ≤ *β_p_* where *β*_1_ to *β_p_* correspond to increasing dose level. This constraint reflects an observation that increasing dose is always associated with lower cell survival fraction, which either increases risk of toxicity or maintains the same risk. The model incorporating these additional constraints is referred to as Knowledge Constrained Lasso, or KC-LASSO.

#### 2.3.3. Investigating Spatial Susceptibility of the Heart to Radiation Damage

##### 1) Digital Subdivision of the Heart

We examined possible dependence of OS on spatial distribution of dose in the heart through digital subdivision of the heart [8]. Two schemes for digitally sub-dividing hearts were utilized: 1) coarse subdivision into four segments, right-superior, left-superior, right-inferior and left-inferior; 2) fine subdivision into 64 segments. To subdivide each heart, we established the smallest rectangular box which fully enclosed the heart structure, oriented along the principal axes of the body (superior-inferior, left-right, and anterior-posterior). In the coarse subdivision the sup-inf and right-left axes were divided with respect to the heart centroid and heart voxels were assigned to their respective segments. In the fine subdivision each of the three sides of the box was divided into four equal parts, and voxels were assigned to respective segments. The fine subdivision was designed to ensure each segment had enough voxels to make DVH analysis possible.

##### 2) Analysis of Coarse Subdivision

Each of the four segments in the coarse subdivision was analyzed using the same methodology as the whole heart DVH. No additional correlations among segments were considered. Results of whole heart and coarse subdivision were used to guide the fine subdivision analysis.

##### 3) Analysis of Fine Subdivision

Fine subdivision analysis was based on the range of doses that were found to be predictive in the whole heart and coarse subdivision analysis. KC-Lasso analysis was performed in the predictive dose range, on each of the 64 heart segments separately. If the p-value associated with a segment was less than 0.05, the segment was added to the cluster of predictive segments. The KC-Lasso analysis was then repeated using the DVH of the entire cluster.

#### 2.3.4. Predicting Cardiac Toxicity Using Previously Developed Model

A previously developed predictive model of radiation induced cardiac toxicity in LA-NSCLC patients [8] was applied to lung SBRT patients in the present study. The model was developed using digital heart subdivision techniques similar to the present work and combined a predictive cluster of segments in the right-superior region of the heart with the DVH based risk assessment formula.

## 3. Results

### 3.1. Patient Characteristics

A total of 197 patients were included in this study. Basic patient characteristics are shown in Table 1.

### 3.2. Patient Specific Covariates

Patient specific covariates which were predictive for OS are shown in Table 2. Higher age (p < 0.001), higher stage (p < 0.001) and squamous cell histology (p = 0.001) were associated with reduced OS. Right lung disease was associated with longer OS (HR = 0.56, p = 0.014).

### 3.3. Dosimetric Analysis

Results of dosimetric analysis and a comparison with corresponding results in LA-NSCLC patients [8] are summarized in Table 3.

#### 3.3.1. Univariate Analysis of the Whole Heart DVH

No dosimetric variables predictive for OS were found in the whole heart DVH for lung SBRT patients. In contrast, high doses to the whole heart were associated with reduced OS in LA-NSCLC patients (*V*_55*Gy*_, HR = 1.04/1%, p = 0.03, reference [8]).

#### 3.3.2. Univariate Analysis of the Digital Subdivision of the Heart into Four Segments

**Figure 1.**
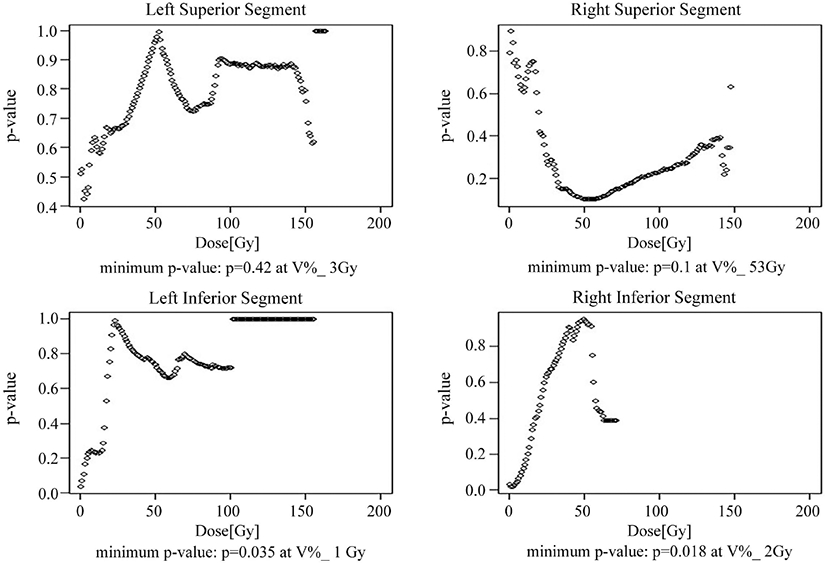
Results of the analysis of lung SBRT patient cohort, using a family of multivariate Cox models, each model with all patient specific covariates and a single *V_D_* index representing heart dosimetry in one of the four segments of the heart (Right-Superior, Left-Superior, Right-Inferior, Left-Inferior). P-values associated with each *V_D_* index are plotted against the dose D (2 Gy Equivalent). All patient specific covariates listed in Table 2 were included in each model fit.

A digital subdivision of the heart into four segments with univariate dose index analysis revealed that *V_D_* indices in the inferior segments of the heart, at doses lower than 5 Gy, were predictive for OS in lung SBRT patients. The lowest p-values were obtained for *V*_2*Gy*_ (HR = 1.009/1%, p = 0.02) in the Right-Inferior segment of the heart, and the *V*_1*Gy*_ (HR = 1.007/1%, p = 0.04) in the Left-Inferior segment of the heart. The dependence of p-values on the selected index is shown in Figure 1. Maximum dose in the Right-Inferior segment of the heart was also predictive for OS (HR = 1.02/Gy, p = 0.02).

In contrast, similar analysis in LA-NSCLC patients showed that high doses to the Right-Superior portion of the heart were predictive for reduced OS (HR = 1.016/1%, p = 0.04, reference [8]). It is noteworthy that the Right-Superior segment of the heart in lung SBRT patients (Figure 1) is the only segment which shows a distinct, pronounced minimum in p-values at high doses (*V*_53*Gy*_, p = 0.1). While the minimum p-value does not reach the level of statistical significance, this pattern is suggestive as it may indicate that the Right-Superior segment of the heart could become a significant predictor of OS in lung SBRT patients if patient numbers in the study were greater.

#### 3.3.3. KC-Lasso Analysis of the Digital Subdivision of the Heart into Four Segments

The KC-Lasso analysis in the *V*_1*Gy*_ - *V*_5*Gy*_ index range yielded a predictive feature in the Right-Inferior segment (HR = 1.01/1%, p = 0.02) and marginally predictive feature in the Left-Inferior segment (HR = 1.01/1%, p = 0.08). Coefficients for both features are listed in Table 3.

#### 3.3.4. KC-Lasso Analysis of the Digital Subdivision of the Heart into 64 Segments

**Figure 2.**
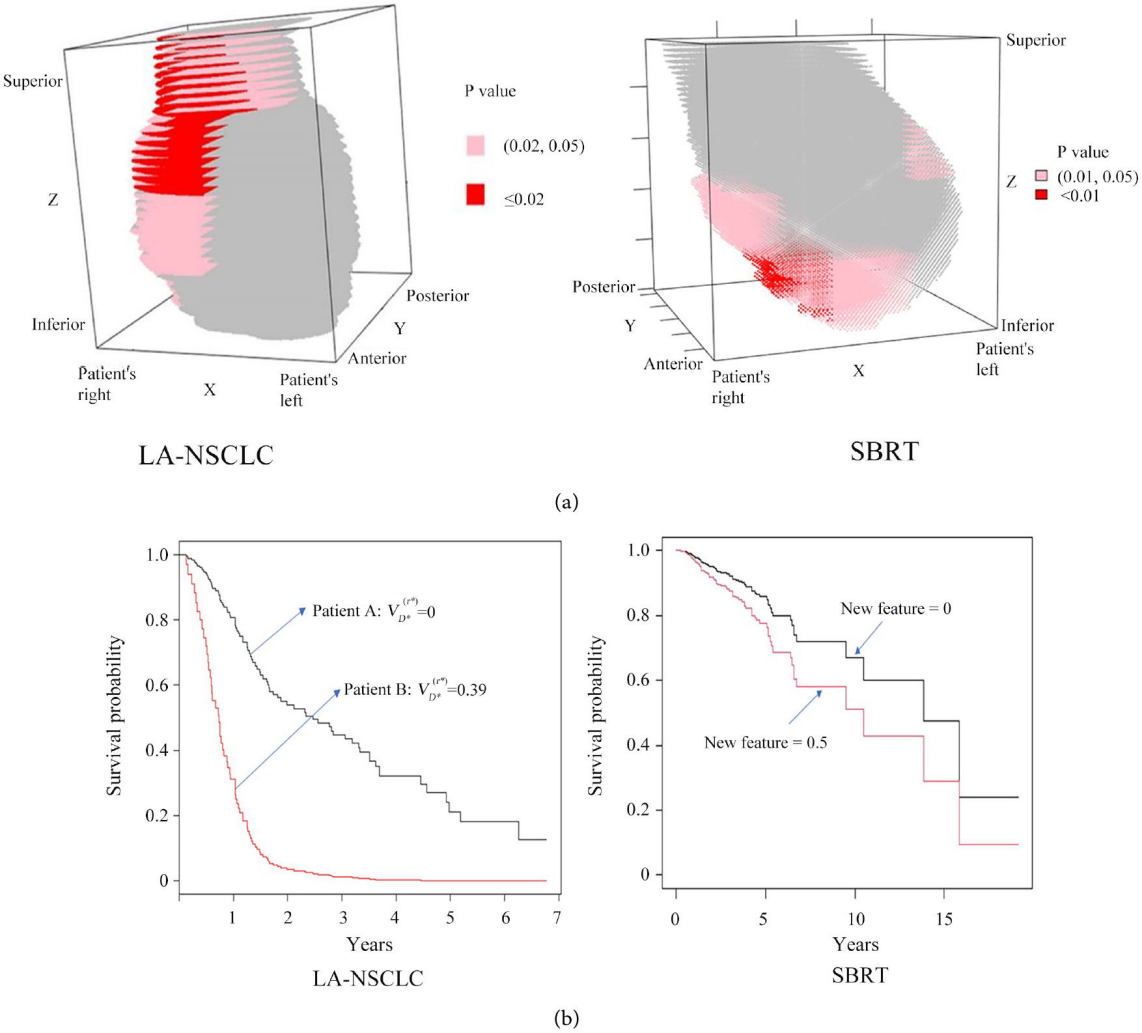
(a) 3D rendering of heart regions predictive for OS in lung SBRT patients (right side, this work) and in LA-NSCLC patients (left side, reference [8]). Light and dark red signifies p-value levels during modelling of clinical significance of individual segments; (b) Survival curves represent effects of irradiating heart regions shown in (a). The LA-NSCLC view shows effects of irradiating 39% of the visualized right-superior region to the dose of 55 Gy or greater; *V*_55*Gy*_ = 39% (reference [8]). The SBRT view shows effects of the KC-LASSO feature shown in Table 3, computed on the colored, inferior heart region, assuming the value of 0.5.
$$\left[h(t)=0.0024*\left(V_{{}_{1Gy}}+V_{{}_{2Gy}}+V_{{}_{3Gy}}+V_{{}_{4Gy}}+V_{{}_{5Gy}}\right)=0.5\right].$$

Digital subdivision of the heart into 64 segments identified a region in the inferior part of the heart which was predictive for OS. The region was formed by 15/64 segments and its location is shown in Figure 2. The best p-value was obtained for a linear combination of *V_D_* indices in the *V*_1*Gy*_ - *V*_5*Gy*_ range (HR = 1.012/1%, p = 0.01). The region can be described as a list of segments: [1, 1, 1], [1, 1, 2], [1, 2, 1], [1, 2, 2], [1, 3, 1], [1, 3, 2], [1, 4, 1], [2, 1, 1], [2, 1, 2], [2, 2, 1], [2, 3, 1], [2, 3, 2], [2, 4, 1], [3, 1, 1], [3, 2, 1], [3, 3, 1], [4, 3, 1], with the reference frame described as [Inferior-Superior, Right-Left, Post-Ant] and each index changing from 1 to 4. Coefficients of the feature are summarized in Table 3.

In contrast, similar subdivision of the heart in LA-NSCLC patients identified a region of similar size, but centered on the Right-Superior region of the heart and predictive for OS when exposed to high doses (*V*_55*Gy*_, HR = 1.043/1%, p < 0.001, reference [8]).

Locations of the two regions, lung SBRT and LA-NSCLC, are compared in Figure 2(a). Survival curves in Figure 2(b) compare the severity of cardiac toxicity in LA-NSCLC and SBRT patients.

#### 3.3.5. Applying LA-NSCLC Model of Cardiac Toxicity to Lung SBRT Patients

The model of cardiac toxicity developed in reference [8] for LA-NSCLC patients predicted no OS reduction in lung SBRT patients because 2 Gy equivalent doses to the Right-Superior portion of the heart were lower than 55 Gy for all patients.

## 4. Discussion

A literature search revealed four studies addressing a possibility of reduced OS due to heart irradiation in lung SBRT. Two studies yielded positive findings [13] [14], and two studies yielded negative findings [15] [16]. Stam *et al*. [13] found an association between non-cancer mortality and the irradiation of Superior Vena Cava or left Atrium in a cohort of 803 patients. Wong *et al*. [14] found an association between OS and the irradiation of Ventricles in a cohort of 189 patients. Tembhekar *et al*. [16] found no association between OS and heart dosimetry in a cohort of 102 patients, and Reshko *et al*. [15] found no association between OS and heart dosimetry in a cohort of 74 patients.

Present findings are qualitatively consistent with the positive findings of Wong *et al*. [14] who found that maximum dose to both Ventricles (combined) was the only dosimetric variable predictive for reduced OS. Our study relied on dose-volume variables as candidate covariates, but we also tested maximum doses in the inferior segments of the heart and found that maximum dose in the Right-Inferior segment was predictive for reduced OS in multivariate analysis (HR = 1.02/Gy, p = 0.02). One should note that dosimetric variables are correlated and it is thus expected that multiple sets of dosimetric variables can be identified as predictors of the same clinical endpoint.

Compared to the positive finding of Stam *et al*. [13], the present study did not find any association between dosimetric variables in the superior segments of the heart and reduced OS in lung SBRT patients. Stam *et al*. used “non-cancer death” as their clinical endpoint and their study had a larger number of patients than the present study (803 patients in Stam *et al*. versus 197 patients in this study). It is interesting to note that p-values in the univariate analysis of the Right-Superior segment of the heart in the present study (Figure 1) show a pronounced minimum at high doses, reaching a minimum value of p = 0.1 at *V*_53*Gy*_. Even though this result is not statistically significant it is suggestive, as the minimum p-value could decline further in a study with larger patient numbers. Considering differences in the study design and patient numbers it is reasonable to say that this study and the study of Stam *et al*. are not contradictory.

Both published studies with negative results, Tembhekar *et al*. [16] and Reshko *et al*. [15], had significantly lower number of patients than the present study, or the two published studies with positive findings. Smaller number of patients can affect statistical significance of any findings which means that these studies cannot be directly compared to the present study.

We compared results of the present study to a previously published study [8] on the association between heart irradiation and reduced OS in locally advanced NSCLC (LA-NSCLC) patients. The comparisons are summarized in Table 3 and Figure 2(a), Figure 2(b). Both studies used similar methods of statistical analysis, including the digital subdivision of the heart to examine spatial patterns of heart irradiation that correlated with OS. A predictive model developed in the LA-NSCLC study [8], when applied to the present data, suggested that heart irradiation during lung SBRT treatments would not lead to OS reduction because doses to the heart were too low, when compared to LA-NSCLC patients. Results of the statistical analysis of lung SBRT patients (Table 3 and Figure 2(a), Figure 2(b)) support predictions of the model. High doses to the right superior segments of the heart were associated with reduced OS in LA-NSCLC patients. In contrast, low doses to the inferior segments of the heart were associated with reduced OS in lung SBRT patients. Survival curves in Figure 2(b) suggest that the OS reduction in LA-NSCLC can be quite severe and occurs within 1 - 2 years after irradiation. In contrast, the reduction in OS of lung SBRT patients is less pronounced and occurs over longer periods of time. These differences suggest different etiologies of cardiac toxicity in both groups of patients.

The etiology of cardiac toxicity in radiation therapy is still poorly understood. The sensitivity of the right-superior structures of the heart to high doses suggests that damage to the conduction system of the heart may be responsible for the most severe cases of cardiac toxicity in radiation therapy. At least one published study was able to associate reduced OS with ECG changes after treatment [9] and most studies associated reduced OS with the irradiation of the superior heart structures to high doses [3] [4] [5] [8] [14]. In studies for which high doses to the heart are common, the most severe damage is likely to dominate statistical analysis and other etiologies of heart damage may remain hidden. These etiologies may become visible to statistical analysis in patient cohorts which receive systematically lower doses to the heart, like lung SBRT patients in the present study.

One could also hypothesize that the association between heart irradiation and OS in lung SBRT may be caused indirectly by the irradiation of the blood. A typical SBRT treatment takes several minutes, while the entire blood volume circulates through the heart in approximately one minute. One can estimate that the entire volume of the blood receives, on average, 1% - 2% of the dose predicted in lower heart chambers. Recent retrospective studies of NSCLC patients support a hypothesis that lymphopenia induced by RT is associated with reduced OS [21] [22] [23] [24]. A recent study by Dupic *et al*. [25] found a correlation between mean lung dose above 5 Gy and reduced OS in lung SBRT. No such correlation could be seen in our study, neither in univariate nor in the multivariate analysis, but this result further suggests that irradiation of the thorax with relatively low doses may be associated with reduced OS. The exact pattern of correlations between low doses and OS may be dependent on the design and the size of a study.

There were several limitations to the present study. This was a retrospective study with a single clinical endpoint of overall survival. The performance status of patients prior to treatment was not available and no information on pre-existing heart condition was recorded. The cause of death was not recorded in patients who died.

## 5. Conclusion

Doses lower than 5 Gy in the inferior segments of the heart may be associated with reduced overall survival in patients treated for lung lesions with SBRT. Stage and histology of the disease, as well as patients’ age, were also associated with overall survival. A comparison of cardiac toxicity patterns in LA-NSCLC patients and lung SBRT patients suggests different etiology of cardiac toxicity in the two groups.

## Figures and Tables

**Table 1.** Patient information, lung SBRT patients (this work). General patient information for lung SBRT patients (this work).

Demographics	
Number of patients	N = 197
Male	N = 95 (48.2%)
Female	N = 102 (51.8%)
Age in years	Mean: 72.7; Range: 41 - 97
Follow up in months	Mean: 40; Median: 28; Range: 2 - 230
Overall Survival in months	Median: 28; Mean: 76.8
Stage	
I (combining IA with IB)	N = 117 (59.4%)
II or III	N = 18 (9.1%)
IV (metastatic lesions)	N = 42 (21.3%)
Histology	
Adenocarcinoma	N = 79 (40.1%)
Squamous cell carcinoma	N = 38 (19.3%)
Lung carcinoma (multiple types)	N = 38 (19.3%)
Non-lung carcinoma (multiple types)	N = 42 (21.3%)
Laterality	
Right	N = 113 (57.4%)
Left	N = 84 (42.6%)
Prescription	
50 Gy in 5 fractions	N = 69 (35%)
48 Gy in 4 fractions	N = 128 (65%)
Lung dosimetry	
Left Lung Dose (2 Gy Equivalent)	Mean: (5.3 ± 6.1) Gy; Range: [0.17 - 33.9] Gy Median: (0.6 ± 1.1) Gy; Range: [0.03 - 8.8] Gy
Right Lung Dose (2 Gy Equivalent)	Mean: (6.0 ± 5.9) Gy; Range: [0.2 - 25.6] Gy Median: (0.8 ± 1.23) Gy; Range: [0.06 - 8.2] Gy
Left Lung V%_20 (2 Gy Equivalent)	Mean: (0.06 ± 0.08); Range: (0 - 0.37)
Right Lung V%_20 (2 Gy Equivalent)	Mean: (0.07 ± 0.08); Range: (0 - 0.34)
Both Lungs V%_20 (2 Gy Equivalent)	Mean: (0.066 ± 0.036); Range: (0.01 - 0.18)
Targets	
PTV Volume (cm^3^)	Mean: (52.1 ± 62.64); Range: [7.2, 427.7]
PTV Effective Radius (cm)	Mean: (2.3 ± 2.5); Range: [1.2, 4.7]

**Table 2.** Patient specific covariates. Patient specific covariates which were determined to be predictive for OS in lung SBRT patients (this work) using multivariate Cox model with Akaike Information Criterion (AIC). Heart dosimetry was excluded at this initial step of the analysis. Hazard ratios are calculated with respect to the reference patient, who is 72.2 years old, presents with Stage 1 primary Adenocarcinoma of the left lung.

Covariate	Hazard Ratio	P
Stage		
Stage 1	Reference	
Stage 2 or 3	HR = 3.6	<0.001
Metastatic	HR = N/A	0.37
Histology		
Lung adenocarcinoma	Reference	
Lung squamous cell carcinoma	HR = 2.7	0.001
Lung carcinoma (multiple types)	HR = N/A	0.07
Metastatic	HR = N/A	0.2
Laterality		
Left	Reference	
Right	HR = 0.56	0.014
Other		
Age	HR = 1.09/year	<0.001

Reference patient: 72.2 years old, stage 1 primary adenocarcinoma of the lung, left laterality.

**Table 3.** Summary of the dependence of OS on dosimetric variables in lung SBRT patients (this work) and in locally advanced NSCLC patients (reference [8]). Summary of the dosimetric analysis for lung SBRT patients (this work) and LA-NSCLC patients (reference [8]). *V*_*D*_ indices are expressed as a percentage of volume receiving dose D, or greater. Hazard Ratio (HR) for a specific patient can be computed using a power law. If *V*_*D*_ = *X*% for a specific patient, and the listed HR = *Y*/1%, then the Hazard Ratio for the specific patient is computed as HR = *Y*^*X*^. The same approach can be used for KC-Lasso features, using *X* obtained from summing the feature for a given patient, with each *V*_*D*_ expressed as a percentage of volume receiving dose D, or greater.

	Lung SBRT (this work)			Locally advanced NSCLC (reference [8])		
Feature	Index	HR	P	Index	HR	p
Whole Heart DVH						
Whole Heart DVH	N/A	N/A	0.2	*V*_55*Gy*_	1.04/1%	**0.03**
Heart subdivision into 4 segments						
Right Superior DVH	N/A	N/A	0.1	*V*_55*Gy*_	1.016/1%	**0.04**
Left Superior DVH	N/A	N/A	0.4	N/A	N/A	0.4
Right Inferior DVH	*V*_2*Gy*_	1.009/1%	**0.02**	N/A	N/A	0.26
Left Inferior DVH	*V*_1*Gy*_	1.007/1%	**0.04**	N/A	N/A	0.85
Right Inferior DVH KC-Lasso	0.0021 * *V*_1*Gy*_ + 0.0021 * *V*_2*Gy*_ + 0.0021 * *V*_3*Gy*_ + 0.0021 * *V*_4*Gy*_ + 0.0021 * *V*_5*Gy*_	1.01/1%	**0.02**	N/A	N/A	N/A
Left Inferior DVH KC-Lasso	0.0016 * *V*_1*Gy*_ + 0.0016 * *V*_2*Gy*_ + 0.0016 * *V*_3*Gy*_ + 0.0016 * *V*_4*Gy*_ + 0.0016 * *V*_5*Gy*_	1.01/1%	0.08	N/A	N/A	N/A
Heart subdivision into 64 segments						
Selected Region DVH KC-Lasso	0.0024 * *V*_1*Gy*_ + 0.0024 * *V*_2*Gy*_ + 0.0024 * *V*_3*Gy*_ + 0.0024 * *V*_4*Gy*_ + 0.0024 * *V*_5*Gy*_	1.012/1%	**0.01**	*V*_55*Gy*_	1.043/1%	**<0.001**
